# Seasonal Variation of Essential Oil Quantity and Quality in Bay Laurel (*Laurus nobilis* L.) Leaves from Montenegro

**DOI:** 10.3390/plants15060923

**Published:** 2026-03-17

**Authors:** Zoran S. Ilić, Ljiljana Stanojević, Lidija Milenković, Aleksandra Milenković, Ljubomir Šunić, Dušica Ilić, Jelena Stanojević, Dragan Cvetković, Dragan Božović, Žarko Kevrešan

**Affiliations:** 1 Faculty of Agriculture, University of Priština in Kosovska Mitrovica, 38219 Lešak, Serbia; lidija.milenkovic@pr.ac.rs (L.M.); ljubomir.sunic@pr.ac.rs (L.Š.); 2 Faculty of Technology, University of Niš, Bulevar Oslobodenja 124, 16000 Leskovac, Serbia; ljiljas76@yahoo.com (L.S.); aleksandra.milenkovic@student.ni.ac.rs (A.M.); dusica.aleksandar@gmail.com (D.I.); jelena_stanojevic@yahoo.com (J.S.); dragancvetkovic1977@yahoo.com (D.C.); 3 Research and Development Institute-Tamiš, Novoseljanski put 33, 26000 Pančevo, Serbia; draganbozovic64@gmail.com; 4 Institute of Food Technology, University of Novi Sad, Bulevar Cara Lazara 1, 21000 Novi Sad, Serbia; zarko.kevresan@fins.uns.ac.rs

**Keywords:** *L. nobilis*, essential oil, GC-MS analysis, 1,8-cineole, antioxidant activity

## Abstract

Seasonal variation is recognized as a key factor affecting the essential oil (EO) yield, chemical composition, and antioxidant activity of *Laurus nobilis* L. from the Montenegro coast, which constituted the focus of this research. The bay essential oil (BEO) yield was higher in summer (2.12%) and autumn (2.03%) than in winter (1.26%) and spring (1.28%). The total number of BEO components, depending on seasonal variability, ranges from 31 (summer) to 34 (winter and spring). 1,8-cineole (eucalyptol) was the major aromatic compound in all seasons, with the highest content recorded in summer (52.4%). Linalool, as the second most abundant component, is present in the autumn harvest (14.1%), while α-terpinyl acetate, as the third most abundant component, is most prevalent in the winter–spring period (9.6–9.7%). Two groups of monoterpenes, namely the oxygen-containing monoterpene derivatives (80.1%), constitute the most abundant components in BEO leaves, as well as monoterpene hydrocarbons (14.3–15.5%). The phenylpropanoids were the least abundant (4.9–6.3%). Stronger antioxidant activity (DPPH) during an incubation time of 20 min was shown by the BEOs isolated from autumn (EC50 value of 1.15 mg/mL). Early autumn (September) represents the optimal harvest time for *L. nobilis* in Montenegro, as they ensure a high essential oil yield and better quality, reflected in a high 1,8-cineole content and stronger antioxidant activity of the oil. These results demonstrate that seasonal variations are key factors regulating the quantity and quality of BEO, providing important information for optimizing harvest strategies for medicinal and industrial purposes.

## 1. Introduction

Native to the Mediterranean region, bay laurel (*Laurus nobilis* L.) is an important aromatic and medicinal plant widely distributed and used worldwide. It is an evergreen plant that grows wild as a shrub or tall tree, with aromatic leaves rich in essential oils (EO). *L. nobilisis* is a prominent culinary herb in Mediterranean cuisine and folk medicine [1]. The leaves possess an astringent and bitter taste, and an aroma comparable to oregano and thyme, with pronounced eucalyptus notes released upon mechanical disruption of the leaves [2].

Bay essential oil (BEO) is widely utilized as a natural ingredient across the food flavoring sector, as well as in aromatherapy, therapeutic massage practices, and cosmetic formulations, and is additionally employed as a fumigant for the protection of stored wheat grains against *Aspergillus flavus*, owing to their pronounced repellent properties [3].

BEO has insecticidal and antifungal properties, making it a natural alternative to synthetic pesticides. Traditionally used by Lebanese farmers as a pest repellent, it is now valued in sustainable agriculture [4]. Incorporating bay essential oil into reduced-salt table olives highlights its capacity to improve product stability while upgrading traditional foods with greater functional and market value [5].

BEO is influenced by environmental and geographical factors, showing clear seasonal variation with maxima in autumn and minima in spring, and higher levels in northern and eastern populations [6]. EOs are present throughout every part of the bay plant. Thus, the fruits (0.88%) and separated stems (0.7%) contain smaller amounts of essential oils than the shoots (1.4–1.5%) and leaves (2.65%) [7,8].

Significant regional chemotypic variability was observed in the chemical composition of the essential oil of this species [1]. Extensive literature evidence indicates that bay essential oil (BEO) comprises numerous volatile constituents, with 1,8-cineole (eucalyptol) serving as the primary constituent in the oils extracted from Croatia, Montenegro, Spain, Italy, Georgia, Greece, Bulgaria, Tunis, Turkey, Algeria, and Palestine [7,8,9,10,11,12,13,14,15,16,17]. Bay leaves from some other regions contained different EO constituents. Thus, β-caryophyllene (10.0) and viridiflorene (12.2) are the main parameters in BEO from France [18], while eugenol (44.13%) is the main component of BEO from China [19].

The last study on leaf BEO from Montenegro identified fifty chemical constituents, with 1,8-cineole (39.4%), linalool (13.9%), α-terpinyl acetate (11.2%), sabinene (6.7%), and methyl eugenol (5.7%) as the most prevalent [8]. Comparative analysis with other reports revealed that 1,8-cineole was also the dominant volatile in BEO from Central Dalmatia, Croatia, representing 45.5% of the composition, while methyl eugenol, α-terpinyl acetate, linalool, and sabinene were also significant components [20]. The major volatile compound of BEO from Mljet Island, South Dalmatia, was 1,8-cineole (49.79–64.94%), followed by α-terpinyl acetate, sabinene, linalool, α-pinene, β-pinene, and α-terpineol [21]. Due to the favorable yield and high 1,8-cineole content, harvesting during the mid-September fruiting stage is considered optimal in the agroecological conditions of Iran [22].

The BEO content and composition of leaves show pronounced seasonal variation, making harvest timing a key factor in optimizing oil yield and target compounds [23].

Montenegro’s Mediterranean coastal climate, characterized by high summer temperatures, pronounced seasonal precipitation patterns, and significant solar radiation, represents an ecologically distinct environment that may drive specific seasonal metabolic responses in *L. nobilis*, making it a relevant model system for studying harvest optimization in the region.

This study aimed to examine the seasonal changes in BEO from Herceg Novi (Montenegro) to identify the optimal harvest period for achieving the highest oil yield and the greatest concentration of key compounds. We hypothesized that EO yield and 1,8-cineole content would peak during the active vegetation period (summer–autumn), driven by enhanced monoterpene synthase activity under high temperature and solar radiation, while cooler dormancy conditions (winter–spring) would favor the accumulation of α-terpinyl acetate, sesquiterpenes, and phenylpropanoids, with corresponding seasonal differences in antioxidant activity reflecting these compositional shifts. Although the research was conducted over a single year and confined to one geographic area, notable seasonal fluctuations in the volatile composition of bay laurel leaves are anticipated.

## 2. Materials and Methods

### 2.1. Study Site

Herceg Novi is located at Latitude: 42°27′11.02″ N Longitude: 18°32′15.00″ E. The city experiences a unique microclimate shaped by its southern orientation, closeness to the sea, limestone-based geology, and surrounding mountains that block the intrusion of cold air masses. The hinterland of Herceg Novi is characterized by a highly rugged, mountainous relief dominated by the Orjen massif (peaks exceeding 1800 m), which descends steeply toward the sea. This area, including the Dobroštica hills (1570 m) and Radoštak (1441 m), forms a limestone barrier that protects the coast from cold air masses, creating a distinct Mediterranean microclimate.

The climate is Mediterranean, with hot, dry summers and mild winters. Herceg Novi has a very high mean annual air temperature, which in 2024 was 17.8 °C, with mean monthly temperatures ranging from 28.1 °C in July to 9.5 °C in January, and an average of about 200 sunny days per year. Temperature fluctuations are generally small, with an average daily temperature oscillation of only about 4 °C. The total annual precipitation amounts to 1969 mm, with the highest rainfall in December (380 mm) and the lowest in July (7.5 mm). Relative air humidity is around 63% in summer and reaches up to 83% in autumn (Table 1).

The mean atmospheric pressure is 1011 mbar. During summer (July and August), the city records an average of 10.7 sunshine hours per day, while the annual average is 6.5 sunshine hours per day. With 2273 h of sunshine, Herceg Novi is one of the sunniest cities in Montenegro. It has a large number of sunny days throughout the year (around 250 days with cloud cover > 2).

Winds are mild and generally do not exceed speeds of 1.2–1.7 m/s. Characteristic winds include *maestral* (westerly wind, mainly in summer), *bura* (northerly wind, mainly in winter), *jugo* (southerly wind, mainly in winter), and *šilok* (southerly wind, blowing throughout the year), as well as less frequent winds such as *grego*, *levanat*, *tramuntana*, *oštrijal*, and *pulenat*.

### 2.2. Plant Material

Leaves of *Laurus nobilis* were collected from five mature, healthy trees, 10–15 years age, growing under uniform conditions at the same location in Herceg Novi, Montenegro (GPS: 42°27′11.02″ N, 18°32′15.00″ E; 50 m above s.l.). Trees were selected based on uniform size, vigor, and canopy development to minimize inter-individual variability. To control for spatial variability within the site, leaves were sampled from the central canopy zone of each tree at a consistent height and orientation. Leaf material from all selected trees was combined at each sampling occasion to form a representative pooled sample per season, as is standard practice in essential oil seasonal variation studies. Sampling was conducted four times during 2024, corresponding to distinct physiological stages of *L. nobilis*: relative dormancy (January), active vegetative growth and flowering (April), fruit development (June), and fruit ripening (September). Dr. Zoran Ilić botanically identified the plant material. After harvesting, leaves were air-dried for 20 days in a well-ventilated room, shielded from direct sunlight, at approximately 22 °C and 60% relative humidity. The average sample per tree was 100 g, and the total sample mass was 0.5 kg for each harvest date. Dried material was stored in a cool, dry location, and a voucher specimen was deposited in the herbarium of the University of Priština in Kosovska Mitrovica (voucher number UKM-1389).

### 2.3. Essential Oil Isolation

Dried *Laurus nobilis* leaves (100 g) were hydrodistilled for 2 h (1:10 *w*/*v*) using a Clevenger apparatus; oils were collected, dried, and stored at 4 °C, and yields (%, *v*/*w*) were determined in triplicate.

### 2.4. GC/MS and GC/FID Analysis

GC/MS analysis was performed on an Agilent Technologies 7890B gas chromatograph equipped with a nonpolar silica capillary column, HP-5MS (5% diphenyl- and 95% dimethyl-polysiloxane, 30 m length × 0.25 mm internal diameter, 0.25 μm stationary phase thickness; Agilent Technologies, Santa Clara, CA, USA), and coupled with an inert, selective 5977A mass detector of the same company. The samples were dissolved in diethyl ether to a concentration of 20 mg/mL. One microliter of the solution prepared was injected into the GC column through a split/splitless inlet set at 220 °C in 40:1 split mode. Helium was used as the carrier gas at a constant flow rate of 1 cm^3^/min. The oven temperature increased from 60 °C to 246 °C at a rate of 3 °C/min. The temperatures of the MSD transfer line, ion source, and quadrupole mass analyzer were set at 300 °C, 230 °C, and 150 °C, respectively. The ionization voltage was 70 eV, and the scanned mass range was *m*/*z* 41–415. GC/FID analysis was carried out under identical experimental conditions as GC/MS. The flows of the carrier gas (He), make-up gas (N_2_), fuel gas (H_2_), and oxidizing gas (Air) were 1 cm^3^/min, 25 cm^3^/min, 30 cm^3^/min, and 400 cm^3^/min, respectively. The temperature of the flame-ionization detector (FID) was set at 300 °C [24].

The identification of the compounds was based on the comparison of their retention indices with those reported in the literature and by comparison of their mass spectra with those from the NIST and Wiley spectral libraries. The retention indices were determined relative to a homologous series of n-alkanes (C8–C25) analyzed under the same chromatographic conditions. The relative percentages of the individual components were calculated from the GC–FID peak areas without the use of correction factors.

### 2.5. Antioxidant Activity

#### 2.5.1. DPPH Assay

Antioxidant activity of the essential oils was assessed using the DPPH assay, with EC_50_ values calculated from absorbance at 517 nm. EC_50_ values (mg/mL), representing the concentration required to reduce the initial DPPH radical concentration by 50%, were calculated. Lower EC_50_ values indicate higher antioxidant activity. All measurements were conducted in triplicate.

#### 2.5.2. FRAP (Ferric Reducing Ability of Plasma) Assay

The *FRAP* assay was additionally used to determine the antioxidant activity of BEO according to the method of Benzie and Strain [25], with certain modifications by Stanojević et al. [26]. The results were expressed as Fe^2+^ equivalents (mg/g EO) based on absorbance at 593 nm [27].

### 2.6. Statistical Analysis

Data are presented as mean ± standard deviation (SD) of three independent analytical replicates (*n* = 3) for EO yield, DPPH, and FRAP measurements. One-way ANOVA was applied to assess significant differences among seasons (*p* < 0.05), with means separated using Duncan’s multiple range test. Data reliability was ensured through rigorous analytical quality control, including external calibration of instruments, use of analytical standards, validated extraction and quantification procedures, and consistent sample preparation protocols.

PCA was performed on all measured variables based on the correlation matrix of standardized data, with components retained using the Kaiser criterion (eigenvalue > 1) and factor loadings ≥ |0.70| considered significant for interpretation.

## 3. Results and Discussion

### 3.1. Bay Essential Oil Yields and Composition

The yields of essential oils ranged between 1.26% (January) and 2.12% (June) by weight. April and September samples had 1.28% and 2.03% (*w*/*w*) volatile oil (Table 2).

**Figure 1:**
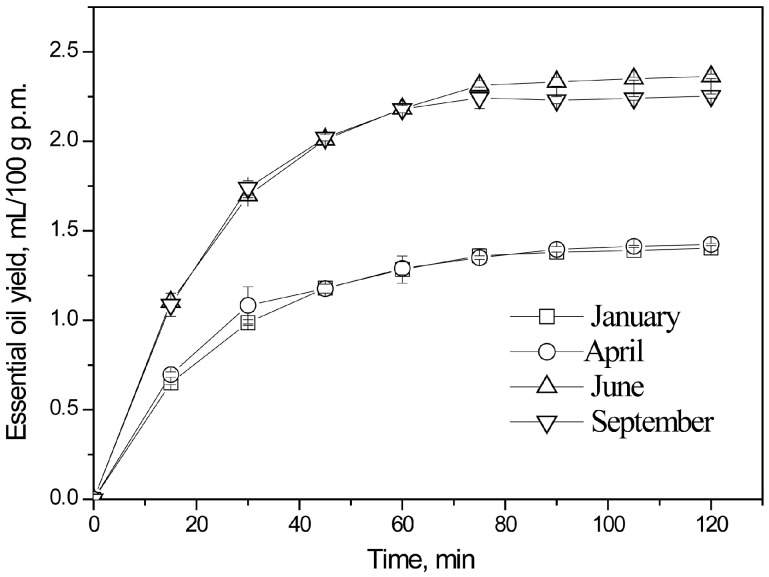
Kinetic study of EO hydrodistillation from bay laurel leaves: impact of harvest timing and extraction length.

Hydrodistillation kinetics of essential oil in bay laurel leaves, as influenced by harvest time and extraction duration, are presented in Figure 1.

Variations in the yield and composition of essential oils (EOs) reported in this and previous studies can result from multiple factors, including collection site, soil properties, climate, harvest time, plant genotype, post-harvest handling, and extraction method.

In Montenegro, the yield of *Laurus nobilis* leaves was 2.65%, similar to our earlier findings [6]. In Croatia, EO content ranged from 0.47% in February to 0.90% in May [20], while in Iran, it varied between 0.8% and 1.5% (*v*/*w*).

Other studies have shown seasonal and processing effects on EO yield. Şekeroğlu et al. [28] reported 2.02–3.02% EO in dried leaves, depending on harvest month (October–June) and drying temperature (35–80 °C), with the highest yields at 35–50 °C in October. In Mediterranean countries, yields were generally lower: Algeria at 1.13% [4] and Morocco at 1.06% [29]. In Tunisia, EO yield ranged from 0.9% to 2.2%, peaking in July across four growth stages [30]. Similarly, in northwestern Iran, yields varied from 0.6% in November to 1.1% in September, depending on developmental stage [31].

Overall, EO content typically increases in early summer, peaks in late July, and declines thereafter [32]. In cultivated trees, the highest EO levels are observed in autumn, while the lowest occur in late spring [33]. The total number of BEO components, depending on seasonal variability, ranges from 31 in summer to 34 in winter and spring. 1,8-cineole (eucalyptol) was the major aromatic compound in all seasons, with the highest content recorded in summer (52.4%). Linalool, as the second most abundant component, is present in the autumn harvest (14.1%), while α-terpinyl acetate, as the third most abundant component, is most prevalent in the winter–spring period (9.6–9.7%), Table 3.

The predominant compounds in BEO leaves are monoterpenes, primarily consisting of oxygenated derivatives (80.1%) and monoterpene hydrocarbons (14.3–15.5%). The phenylpropanoids were the least abundant (4.9–6.3%) (Table 3).

Some components, such as spathulenol, caryophyllene oxide, and 5-neo-Cedrano, are not present in the BEO of leaves from the summer and autumn harvests. Only one component (exo-2-Hydroxycineole acetate) is not present in the leaves from the winter and spring harvests (Table 3).

Climate plays a crucial role in the biosynthesis of volatile oils: warmer and drier conditions enhance the accumulation of monoterpenes for short-term defense against stress and herbivores, whereas cooler, more humid environments favor the production of sesquiterpenes and phenylpropanoids, supporting long-term protection and reproductive processes [36].

Similarly, we encounter the results of our research in studies from Croatia [21]. The BEO was composed of monoterpenes (95.56–99.28%), with minor amounts of other compounds. The dominant volatile was 1,8-cineole (49.79–64.94%), followed by α-terpinyl acetate (7.14–11.96%), sabinene (3.16–9.01%), and linalool (1.77–8.03%) [21].

In a previous study, Ilić et al. [8] reported that BEO leaves are dominated by two groups of monoterpenes. Oxygenated monoterpene derivatives accounted for 72.2%, with 1,8-cineole (39.4%) and linalool (13.9%) as the principal compounds, while monoterpene hydrocarbons made up 15.6%, primarily represented by sabinene (6.7%). Phenylpropanoids contributed 7.8% of the total composition [8].

In Montenegro, research findings indicate that the most abundant components in BEO from Skadar Lake were 1,8-cineol (35.1%), α-terpinyl acetate (10.4%), and linalool (7.6%) [37].

**Figure 2:**
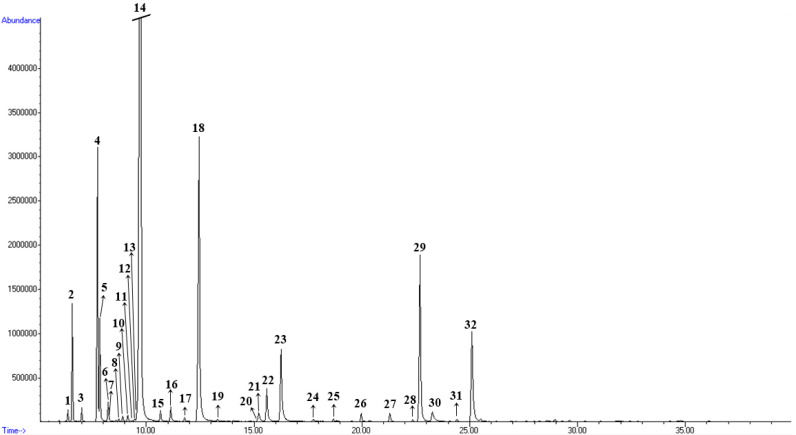
GC/MS chromatogram of bay essential oil from autumn leaves harvest. The names of the compounds are listed in Table 1.

Studies from countries including Bulgaria, Argentina, Albania, Iran, Turkey, and Serbia [38] have identified 1,8-cineole, α-terpinyl acetate, linalool, and sabinene as the main constituents of BEO. In Moroccan (45.01%) and Iranian (30.80–40.25%) bay leaves, 1,8-cineole was similarly the predominant compound [22,28]. Evidence from Lebanon shows that bay leaves possess distinct essential oil profiles, with significant variation in major components such as 1,8-cineole, sabinene, and α-terpinyl acetate, influenced largely by altitude and climate. In Serbian bay leaves, 1,8-cineole (eucalyptol) dominates at 40.51%, followed by α-terpineol (15.46%), while α-pinene occurs at much lower levels (4.45%) [38]. These results highlight the presence of geographically defined chemotypes with potential functional significance [1]. The GC-MS chromatogram, with the compound identified numbered above each peak according to the order of their elution on the HP-5MS column, is given in Figure 2.

Seasonal changes strongly affect the yield and composition of *Laurus nobilis* essential oil. In Iran, 1,8-cineole levels peaked in June (40.25%) and remained high through September, coinciding with full flowering to fruit-bearing stages, while winter months showed lower concentrations [31].

Similar patterns have been observed in Tunisia [30] and Portugal [39], where EO composition shifts with season rather than location, with oxygenated compounds increasing during the rainy season. Similarly, research on *Laurus nobilis* in Portugal demonstrated that peak EO yields, typically at the end of August, coincide with lower hydrocarbon percentages and higher levels of oxygenated compounds, supporting our observations.

Overall, these seasonal differences highlight the strong influence of environmental factors such as temperature, humidity, and light on essential oil production and accumulation. Winter conditions tend to favor the synthesis of monoterpenes and sesquiterpenes, whereas the rainy season promotes oxygenated derivatives. These findings highlight the importance of harvest timing to optimize both oil yield and bioactive content for commercial and pharmacological applications.

### 3.2. Antioxidant Activity

Bay essential oils extracted from autumn-harvested leaves (EC_50_ 1.15 mg/mL) exhibited stronger DPPH antioxidant activity over 20 min compared to summer-harvested oils (EC_50_ 2.21 mg/mL). Additionally, the summer harvest showed the lowest FRAP value, measuring 19.04 mg Fe^2+^/g EO among all collection periods (Table 4).

**Figure 3:**
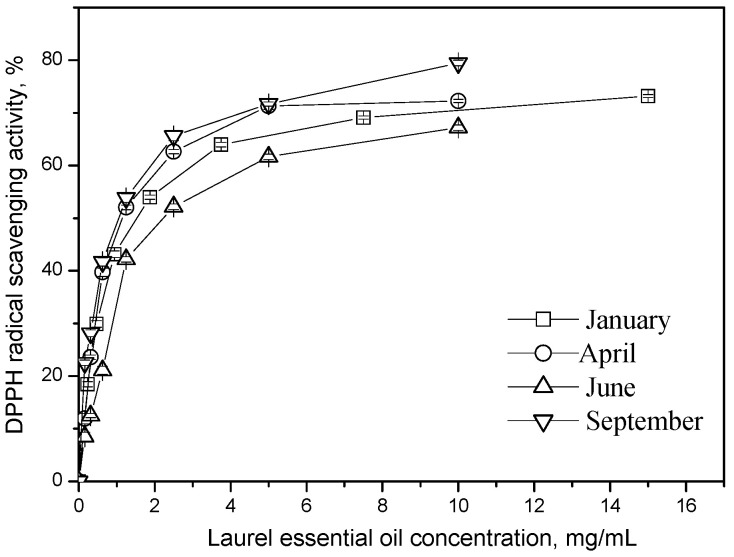
Harvest time effect on DPPH radical neutralization activity of bay leaves essential oil.

Early autumn (September) represents the optimal harvest time for bay laurel in Montenegro, as it ensures a high yield of essential oil and better quality, reflected in a high 1,8-cineole content. Additionally, essential oils obtained from leaves during this harvest period exhibited stronger antioxidant activity (DPPH) (Figure 3).

Consistent with previously reported data, the antioxidant activity of BEO also exhibits seasonal variations, with the highest activity recorded in spring (268.6 µg/mL) and the lowest in winter (702.1 µg/mL) in Iranian laurel [40], a pattern partially consistent with our findings.

These results demonstrate that seasonal variations are key factors regulating the quantity and quality of BEO, providing important information for optimizing harvest strategies for medicinal and industrial purposes.

The observed seasonal pattern in antioxidant activity can be partially explained by the corresponding variation in EO composition. The strongest DPPH radical scavenging activity recorded in autumn coincides with elevated levels of linalool (14.1%), eugenol (1.2%), and methyleugenol (4.5%)—compounds with well-documented radical scavenging properties attributed to their hydroxyl groups and conjugated double bond systems [22,40]. Conversely, summer oils, despite containing the highest 1,8-cineole content (52.4%), exhibited the weakest DPPH activity, suggesting that 1,8-cineole contributes minimally to radical scavenging, consistent with its non-phenolic monoterpene ether structure [1]. The highest FRAP values recorded in winter and spring similarly correspond to elevated phenylpropanoid content, particularly methyl eugenol (5.0–5.1%). Collectively, these findings suggest that phenylpropanoids, rather than the dominant monoterpene 1,8-cineole, are the primary drivers of antioxidant activity in BEO, consistent with previous reports on laurel essential oil bioactivity [8,40].

#### Principal Component Analysis (PCA)

PCA was applied to all measured variables (EO yield, DPPH, FRAP, and individual EO components) to explore multivariate relationships among seasons. The first two principal components (PC1 and PC2) cumulatively explained 94.2% of total variance (76.2% and 18.0%, respectively), providing a comprehensive representation of the data structure.

**Figure 4:**
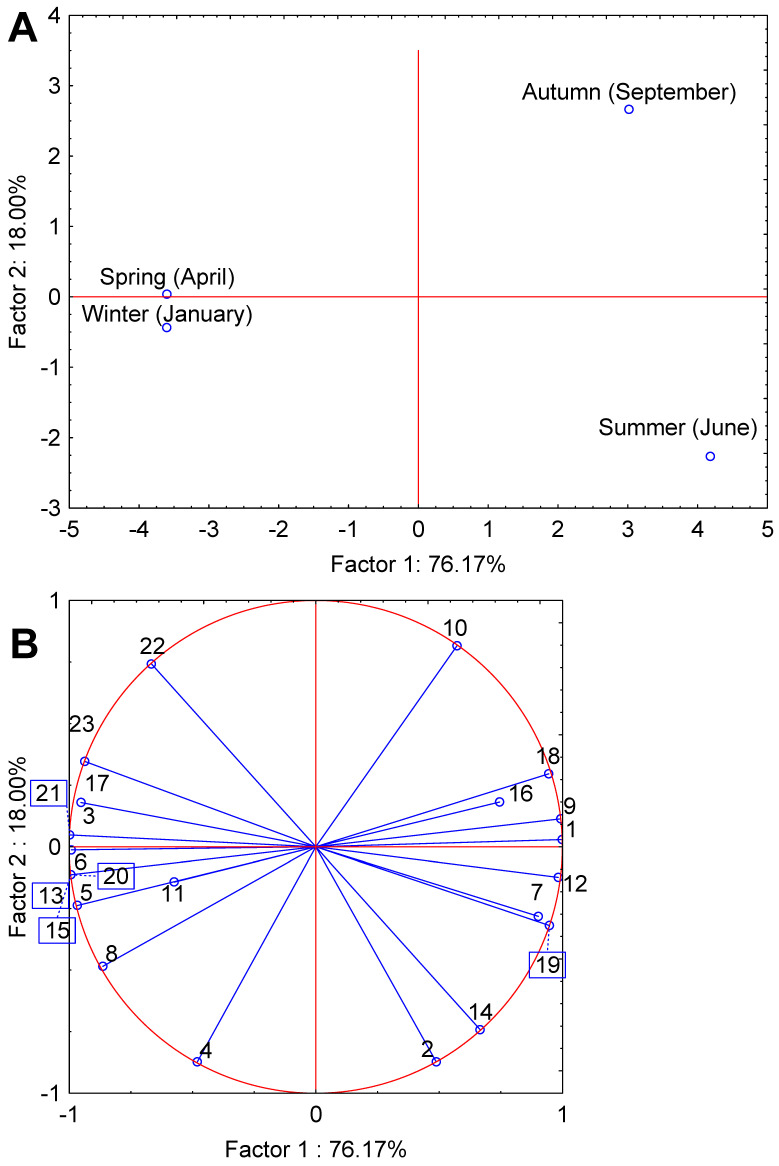
Principal component analysis (PCA) biplots for bay laurel showing season-specific quality responses to harvest time. Projection of cases (**A**) and variables (**B**) on the factor plane. Variables: 1—Yield (cm^3^/100 g p.m.), 2—DPPH EC50 (mg/mL), 3—FRAP (mg Fe^2+^/g d.e.), 4—α-Thujene, 5—α-Pinene, 6—Camphene, 7—Sabinene, 8—β-Pinene, 9—Myrcene; 10—δ-3-Carene, 11—α-Terpinene, 12—1,8-Cineole, 13—γ-Terpinene, 14—cis-Sabinene hydrate, 15—Terpinolene, 16—Linalool, 17—Terpinen-4-ol, 18—α-Terpineol, 19—Bornyl acetate, 20—Dihydrocarveol acetate, 21—α-Terpinyl acetate, 22—Eugenol, 23—Methyl eugenol.

The projection of variables onto the PC1 × PC2 factor plane (Figure 4) revealed that EO yield, 1,8-cineole, Myrcene, Sabinene, α-Terpineol, and Bornyl acetate were strongly and positively associated with PC1, while FRAP, α-Terpinyl acetate, Camphene, α-Pinene, and Terpinen-4-ol showed strong negative loadings on the same axis. DPPH EC_50_ and α-Thujene loaded predominantly on PC2 (negatively), whereas δ-3-Carene, Eugenol, and Methyleugenol were positively associated with PC2.

## 4. Conclusions

To identify the ideal harvest period for maximum yield and quality of essential oil, *Laurus nobilis* leaves were sampled at the midpoint of each season—spring, summer, autumn, and winter. Statistical analysis confirmed that the timing of harvest significantly influenced BEO production. The results showed that both essential oil composition and antioxidant activity varied throughout the year, influenced by seasonal climatic conditions. Between 31 and 34 constituents in the essential oils were identified at different harvest times, with 1,8-cineole consistently detected as the predominant compound across all seasons. Considering the high oil yield, the dominance of 1,8-cineole, and the strong antioxidant activity, autumn (September) was identified as the most favorable period for harvesting.

Considering the high oil yield, the dominance of 1,8-cineole, the elevated phenylpropanoid content, and the strong antioxidant activity, particularly the superior DPPH radical scavenging capacity, autumn (September) was identified as the most favorable period for harvesting.

Nevertheless, several limitations of the present study should be acknowledged. The dataset is confined to a single year (2024) and a single location with five sampled trees, which may limit the generalizability of the findings with respect to inter-annual climatic variability and the broader genetic diversity of the species. Additionally, the absence of soil physicochemical characterization restricts the evaluation of potential edaphic influences on EO composition. Future studies should incorporate multi-year datasets, multiple locations across the Montenegro coast, and soil analysis to confirm the observed seasonal patterns and determine whether similar trends occur across different Mediterranean chemotypes of *L. nobilis.*

## Figures and Tables

**Table 1:** Basic climatic parameters for Herceg Novi in 2024 https://www.meteo.co.me/doc/godisnjaci/Godi%C5%A1anjak%202024.pdf (accessed on 9 March 2026).

Month	Temperature (°C)			Precipitation (mm)	Relative Humidity %	Sunshine h	Cloudiness < 2	Wind Speed (m/s)
	Max	Min	Average					
I	15	6.3	9.5	136.3	77	72.2	10	1.8
II	17.7	8.4	12.1	205.7	77	116.4	8	-
III	18.4	9.4	13.1	276.1	78	165.1	5	2.0
IV	22.8	11.3	16.1	89.5	73	229.0	9	1.7
V	25.2	15.1	19.9	182.0	78	231.5	3	1.6
VI	30.7	19.6	24.8	118.4	72	298.2	14	1.5
VII	34.5	22.2	28.1	7.5	61	360.9	23	-
VIII	34.8	22.5	27.8	96.3	61	293.7	17	1.6
IX	27.7	17.2	21.3	282.8	74	190.9	-	1.5
X	24.1	14.6	18.4	93.5	83	172.9	10	1.5
XI	18.8	8.4	12.4	101.7	75	92.4	9	-
XII	14.2	7.3	10.0	379.7	81	49.4	6	1.6
Year Average	23.7	13.5	17.8	1969.7	74	2273	114	

**Table 2:** Yield of bay essential oil leaves obtained after 120 min of hydro-distillation.

Season	g/100 g p.m. * % (*v*/*w*)
Winter (January)	1.261 ± 0.010 ^c^
Spring (April)	1.280 ± 0.006 ^c^
Summer (June)	2.128 ± 0.015 ^a^
Autumn (September)	2.030 ± 0.013 ^b^

* p.m.—plant material; Values followed by different letters are significantly different at *p* < 0.05.

**Table 3:** Seasonal variation of leaves bay essential oil composition.

No.	*t*_ret_, min	Compound	RI^exp^	RI^lit^	Method of Identification	Content %			
						Winter	Spring	Summer	Autumn
1.	6.37	α-Thujene	921	924 ^a^	RI, MS	0.3	0.3	0.3	0.2
2.	6.57	α-Pinene	929	932 ^a^	RI, MS	3.3	3.2	2.7	2.6
3.	7.01	Camphene	944	946 ^a^	RI, MS, Co-I	0.4	0.4	0.3	0.3
4.	7.73	Sabinene	969	969 ^a^	RI, MS	6.4	6.0	7.1	6.7
5.	7.85	β-Pinene	973	974 ^a^	RI, MS	3.3	3.2	3.0	2.8
6.	8.24	Myrcene	986	988 ^a^	RI, MS	0.5	0.5	0.8	0.8
7.	8.27	dehydro-1,8-Cineole	987	988 ^a^	RI, MS	tr	tr	tr	tr
8.	8.73	α-Phellandrene	1003	1002 ^a^	RI, MS	tr	tr	tr	tr
9.	8.91	δ-3-Carene	1007	1008 ^a^	RI, MS	0.1	0.1	0.2	0.1
10.	9.14	α-Terpinene	1013	1014 ^a^	RI, MS	0.3	0.2	0.2	0.2
11.	9.35	*p*-Cymene	1019	1020 ^a^	RI, MS	tr	tr	tr	tr
12.	9.46	*o*-Cymene	1024	1022 ^a^	RI, MS	0.2	0.3	tr	tr
13.	9.59	Limonene	1025	1024 ^a^	RI, MS, Co-I	tr	tr	tr	tr
14.	9.72	1,8-Cineole	1029	1026 ^a^	RI, MS, Co-I	47.5	48.2	52.4	51.0
15.	10.66	γ-Terpinene	1054	1054 ^a^	RI, MS, Co-I	0.5	0.5	0.3	0.3
16.	11.13	*cis*-Sabinene hydrate	1067	1065 ^a^	RI, MS	0.4	0.4	0.5	0.4
17.	11.79	Terpinolene	1084	1086 ^a^	RI, MS	0.2	0.2	0.1	0.1
18.	12.45	Linalool	1102	1095 ^a^	RI, MS, Co-I	13.7	12.9	13.9	14.1
19.	13.30	dehydro-Sabina ketone	1122	1117 ^a^	RI, MS	tr	tr	tr	tr
20.	15.19	Borneol	1168	1165 ^a^	RI, MS, Co-I	tr	0.7	tr	tr
21.	15.24	δ-Terpineol	1169	1162 ^a^	RI, MS	0.6	tr	0.5	0.6
22.	15.62	Terpinen-4-ol	1178	1174 ^a^	RI, MS	2.0	2.4	1.0	1.4
23.	16.25	α-Terpineol	1193	1186 ^a^	RI, MS	3.1	3.2	3.5	3.6
24.	17.73	*cis*-Sabinene hydrate acetate	1228	1219 ^a^	RI, MS	tr	tr	tr	0.2
25.	18.68	*trans*-Sabinene hydrate acetate	1250	1253 ^a^	RI, MS	0.1	0.1	0.1	0.1
26.	19.97	Bornyl acetate	1280	1284 ^a^	RI, MS	0.2	0.2	0.4	0.3
27.	21.30	Dihydro carveol acetate	1312	1306 ^a^	RI, MS	0.5	0.5	0.4	0.4
28.	22.34	exo-2-Hydroxycineole acetate	1337	1344 ^b^	RI, MS	-	-	0.2	tr
29.	22.71	α-Terpinyl acetate	1345	1346 ^a^	RI, MS	9.6	9.7	7.2	7.7
30.	23.27	Eugenol	1359	1356 ^a^	RI, MS, Co-I	1.2	1.2	1.0	1.2
31.	24.43	β-Elemene	1387	1389 ^a^	RI, MS	tr	tr	-	0.1
32.	25.11	Methyl eugenol	1403	1403 ^a^	RI, MS	5.0	5.1	3.9	4.5
33.	31.90	Spathulenol	1575	1577 ^a^	RI, MS	0.2	0.2	-	-
34.	31.99	Caryophyllene oxide	1578	1582 ^a^	RI, MS	0.2	0.2	-	-
35.	36.08	5-neo-Cedranol	1688	1684 ^a^	RI, MS	0.2	0.2	-	-
	Total identified (%)					100.0	100.0	100.0	100.0
	Grouped components (%)								
	Monoterpene hydrocarbons (1–6, 8–13, 15, 17)					15.5	14.9	15.0	14.3
	Oxygen-containing monoterpenes (7, 14, 16, 18–28)					77.7	78.2	80.1	80.0
	Sesquiterpene hydrocarbons (30)					tr	tr	-	0.1
	Oxygen-containing sesquiterpenes (32–34)					0.6	0.6	-	-
	Phenylpropanoids (29, 31)					6.2	6.3	4.9	5.7
	Others (-)					-	-	-	-

*t*_ret._: Retention time; RI^lit^ (the retention index) was determined according to the different references (a,b). ^a^ [34] Adams, R.P. Identification of essential oil components by gas chromatography/mass spectrometry, Carol Stream, Allured Publishing Co., Illinois, USA, 2007. ^b^ [35] Benkaci-Ali, F.; Baaliouamer, A.; Meklati, B.Y.; Chemat, F. Chemical composition of seed essential oils from Algerian *Nigella sativa* extracted by microwave and hydrodistillation. *Flavour Fragr. J.* **2007**, *22*, 2, 148–153. https://doi.org/10.1002/ffj.1773. RI^exp^: Experimentally determined retention indices using a homologous series of *n*-alkanes (C_8_–C_20_) on the HP-5MS column. MS: constituent identified by mass-spectra comparison; RI: constituent identified by retention index matching; Co-I: constituent identity confirmed by GC co-injection of an authentic sample; tr = trace amount (<0.05%).

**Table 4:** Seasonal variation of bay leaves essential oil antioxidant activity.

Season	DPPH EC_50_ (mg/mL), 20 min Incubation	FRAP (mg EFe^2+^/g EO)
Winter (January)	1.410 ± 0.156 ^b^	52.281 ± 0.065 ^a^
Spring (April)	1.330 ± 0.019 ^b^	46.94 ± 0.048 ^b^
Summer (June)	2.209 ± 0.027 ^a^	19.04 ± 0.083 ^d^
Autumn (September)	1.156 ± 0.028 ^c^	23.62 ± 0.083 ^c^

Values followed by different letters are significantly different at *p* < 0.05.

## Data Availability

All the data are available in the manuscript file.
